# Associations between Multimodal Fitness Assessments and Rowing Ergometer Performance in Collegiate Female Athletes

**DOI:** 10.3390/sports8100136

**Published:** 2020-10-15

**Authors:** Clifton J. Holmes, Bjoern Hornikel, Katherine Sullivan, Michael V. Fedewa

**Affiliations:** 1 Program in Physical Therapy, Washington University School of Medicine, St. Louis, MO 63110, USA; 2 Department of Kinesiology, University of Alabama, Tuscaloosa, AL 35487, USA; bhornikel@crimson.ua.edu (B.H.); ksullivan8@crimson.ua.edu (K.S.); mvfedewa@ua.edu (M.V.F.)

**Keywords:** critical power, 3-min all-out, maximal oxygen uptake, aerobic capacity, anaerobic power

## Abstract

The purpose was to examine the association of critical power from a three-minute all-out row (CP_3-min_) and peak power from a one-stroke maximum test (1-Stroke) with laboratory-based fitness assessments (peak oxygen consumption [$\dot{V}O$_2peak_] and Wingate anaerobic test [WAnT]) and 6000 m (6K) and 2000 m (2K) rowing ergometer performance. Thirty-one female collegiate rowers (20.2 ± 1.1 years, 70.9 ± 6.9 kg, and 172.2 ± 4.8 cm) participated in fitness and rowing performance testing. Pearson’s correlations, linear regression, and Cohen’s *q* were used to determine statistical relationships. Absolute $\dot{V}O$_2peak_ values displayed significant correlations with 6K_total_ (−0.68), 6K_split_ (−0.68), 2K_total_ (−0.64), and 2K_split_ (−0.43). Relative $\dot{V}O$_2peak_ displayed significant correlations with 6K_total_ (−0.36), and 6K_split_ (−0.37). CP_3-min_ demonstrated significant correlations with 6K_total_ (−0.62), 6K_split_ (−0.62), 2K_total_ (−0.61), and 2K_split_ (−0.99). For 2K_split_, a significant difference was observed between relative $\dot{V}O$_2peak_ and CP_3-min_ correlations with a “large” effect size (*q* = 2.367). Furthermore, 1-Stroke showed significant associations with 6K_total_ (−0.63), 6K_split_ (−0.63), 2K_total_ (−0.62), and 2K_split_ (−0.44), while WAnT produced non-significant correlations. Absolute $\dot{V}O$_2peak_ CP_3-min_ accounted for significant proportions of variance observed with performance measures (*p* < 0.05). Practitioners should consider incorporating CP_3-min_ and 1-Stroke as additional tests for gauging rowing performance.

## 1. Introduction

Successful 2000 m (2K) rowing ability at an elite level requires high degrees of both aerobic and anaerobic power, causing strain on the cardiorespiratory system and the skeletal muscles involved [1]. While there is general agreement that multiple physiological, psychological, and technical factors contribute to performance, the extent to which they do so has rarely been quantified [2]. A major reason for this is that competitive races consist of both on-water and team dynamics that are impractical to do year-round and difficult to replicate on-land. As a result, attempts have been made to create standardized testing and research methods to predict on-water performance.

One commonly measured adaptation to aerobic training is an increase in maximal oxygen uptake ($\dot{V}O$_2max_). High $\dot{V}O$_2max_ has been linked to success in endurance-based sporting events, generally due to athletes with high $\dot{V}O$_2max_ also having a high anaerobic threshold, allowing them a greater work capacity than competitors. Previous literature has established the aerobic nature of rowing and the strong association displayed with $\dot{V}O$_2max_ values [2,3,4]. Anaerobic power is also important, specifically during the start and final “leg” of a race [5]. The Wingate anaerobic test (WAnT) is arguably the most widely used test of anaerobic fitness, and has been established as a valid measure of anaerobic power output during maximal short-term cycling and arm cranking exercise [6]. Unfortunately, traditional $\dot{V}O$_2max_ and WAnT tests are performed in laboratory settings requiring long durations, expensive equipment, and knowledgeable technicians, which may be too impractical for coaches overseeing large teams with time and budget limitations, commonly seen at the collegiate level.

Critical power (CP) is the asymptote of the hyperbolic relationship between intensity and the duration that a certain power output can be sustained [7]. CP is based on the physiological thresholds encountered with incremental exercise progressing towards maximal exertion. It represents the highest aerobic power output that can be sustained without exhaustion for approximately 30 min [8,9]. Strong associations between the CP and rowing performance have been displayed in the literature [10,11], but traditional testing requires multiple exhaustive bouts of exercise. To increase time-efficiency, the 3-min all-out cycle test was established as an alternative to derive CP values [12]. While investigating different modalities to implement these procedures, Cheng et al. [13] found that a single 3-min all-out test on a rowing ergometer is highly correlated with the results produced by traditional measures of CP; however, no measures of rowing performance were taken for comparison [13].

The ability to display a high level of anaerobic power requires both muscular strength and power to produce effective propulsive forces during each stroke of a race [14]. Coaches and other practitioners have explored using alternative methods more specific to rowing like the “Max Power Test”, consisting of 30 s of maximum effort strokes usually attained in 10 strokes or less [15,16]. A more novel method utilized is the one-stroke maximum test (1-Stroke), which has been used in place of 10-stroke max power tests; however, appropriate research evaluating the efficacy of these anaerobic power assessments is lacking.

The Concept II rowing ergometer has become a staple of training and testing for rowers at all competitive levels, with time-trials being commonly used to track improvements. However, previous research has demonstrated that the 2K may not be strongly related to on-water rowing performance [17]. Because of the multiple physiological, psychological, and technical factors associated with rowing competition, a battery of tests to assess strengths and weaknesses of the athletes are needed for practitioners to accurately predict future success when on-water training is impossible. Therefore, the aim of the current study was to examine the associations of CP from a three-minute all-out row (CP_3-min_) and peak power from the 1-Stroke with validated laboratory-based fitness assessments ($\dot{V}O$_2peak_ and WAnT) and with 6K and 2K rowing ergometer performance. Based on previous findings, we hypothesized that CP_3-min_ would show a significant relationship with $\dot{V}O$_2peak_ from a graded exercise test [10]. It was also hypothesized that athletes with higher CP_3-min_ and 1-Stroke power outputs would produce faster 6K and 2K times through the ability to sustain higher work outputs for the duration of the time-trials.

## 2. Methods

### 2.1. Participants

Thirty-one female NCAA Division I collegiate rowers (age: 20.2 ± 1.1 years, body mass: 70.9 ± 6.9 kg, height: 172.2 ± 4.8 cm, and body fat 24.6 ± 6.0%) were recruited from the University of Alabama (Tuscaloosa, AL, USA) women’s rowing team to participate in this study. The cohort consisted of 4 freshmen, 8 sophomores, 8 juniors, and 11 seniors. All testing took place prior to the start of the spring 2020 competitive racing season. The University’s physicians and team athletic trainers medically cleared all athletes before performing any rowing-related activities. Written informed consent was obtained from each participant. This project was approved by the University of Alabama’s Institutional Review Board (IRB #18-11-1776) and conformed to the Declaration of Helsinki.

### 2.2. Procedures

#### 2.2.1. Initial Screening

The first session took place at the University of Alabama’s Exercise Physiology laboratory, while all subsequent sessions were completed at the rowing team’s training facility. Participants reported to the laboratory between 6:00 and 9:00 AM having refrained from eating or drinking anything other than water 3 h before arrival. Nude body mass was measured (to the nearest 0.1 kg) with a calibrated digital scale (Tanita BWB-800, Tanita©, Arlington Heights, IL, USA), and standing height was measured (to the nearest 0.1 cm) with a stadiometer (SECA 213, Seca Ltd., Hamburg, Germany). Two measurements (within 2 mm of each other) of SKF thickness were taken using calibrated skinfold calipers (Lange Skinfold Caliper, Seko, Cambridge, MD, USA) across seven standard sites on the right side of the body. Percent body fat was estimated using the Brozek equation [18,19].

#### 2.2.2. Graded Exercise Testing

Following SKFs, participants performed a maximal graded exercise test (GXT) on a motorized treadmill until volitional fatigue to determine $\dot{V}O$_2peak_. A treadmill-based protocol was implemented at the head coach’s request in order to gauge the athletes’ aerobic fitness levels independent of technical rowing ability. Previous research has demonstrated significant correlations between treadmill and rowing ergometer incremental testing, with treadmill testing producing higher oxygen uptake values [20]. GXT procedures were modified from the Astrand Treadmill Test designed to monitor athletic populations [21,22]. After a brief warm-up, treadmill speed was increased and remained constant (5.0 mph) and grade increased gradually (2.5% incline) every 2 min until the participant reached volitional fatigue. Rating of perceived exertion (RPE) was measured during the final 30 s of each exercise stage using the Borg 6–20 scale [23]. During the treadmill test, participants ran continuously while breathing through a special mouthpiece to measure oxygen uptake. Oxygen uptake was analyzed using open-circuit spirometry with the Parvomedics Truemax 2400 computerized metabolic system (Parvomedics, Salt Lake City, UT, USA) that was calibrated before testing. Peak oxygen uptakes were calculated as the highest 20-s average achieved during the GXT. Both absolute (L/min) and relative (mL/kg/min) $\dot{V}O$_2peak_ were utilized in the statistical analysis.

#### 2.2.3. 1-Stroke Maximum Test

A minimum of 72 h of recovery was given before the second session, where athletes performed anaerobic power testing consisting of the 1-Stroke followed by the WAnT. The rowing team’s training facility contained an area with multiple rowing ergometers (Concept II Inc., Morrisville, VT, USA) that allowed for large scale testing. Testing took place during the team’s afternoon training session between 2:00 and 4:00 PM. The athletes were split into two groups, and within each group, the athletes performed the testing procedures simultaneously. The participants warmed up for 10 min with a low-intensity row with 2–3 “hard” stroke efforts interspersed through the 10 min. Each participant performed two 1-stroke maximum trials. The highest power, in watts, seen for either trial was recorded on a data sheet as the Peak Power. The athletes were given between 90 and 180 s of rest between each of the trials. The athletes were instructed to grab the ergometer handle and position themselves in a fully compressed ready position. When the participant signaled that they were ready, the researcher gave the command “READY, GO”. At that time, the athlete pulled as hard as possible, trying to achieve full extension with the stroke. Once the trial was completed, the participant rested 90–180 s before repeating the same procedure for the second stroke.

#### 2.2.4. Wingate Anaerobic Test

Upon the completion of the 1-Stroke assessment, participants rested for a minimum of 10 min and then proceeded to complete the WAnT. The WAnT was performed on a Monark 894e cycle ergometer (Cykelfabriken Monark AB, Varberg, Sweden) equipped with a 1.0-kg-resistance basket and a photoelectric sensor to record the flywheel revolutions. As with the GXT, the cycle WAnT was implemented at the head coach’s request in order to gauge the athletes’ anaerobic fitness levels independent of technical rowing ability. Before initiation of the WAnT, athletes weighed in and the resistance placed on the basket for each participant was 7.5% of body mass [24,25]. At the start of the test, participants were instructed to begin pedaling against no resistance to reach maximal rpm. The athletes were instructed to achieve maximal physical exertion, citing the RPE scale used during the GXT so athletes had a proper understanding of the level of effort required. At the end of the 5-s countdown, the researcher gave a “GO” command and simultaneously released the resistance basket; data collection began subsequently, ending after 30 s. Each participant, while remaining seated, pedaled at maximal speed for the duration of the test without any attempt to conserve energy.

#### 2.2.5. Three-Minute All-Out Row

A minimum of 72 h of recovery was given before the third session, where athletes performed the 3-min all-out row. Testing took place during the team’s afternoon training session between 2:00 and 4:00 PM. The following procedures were based on previous research [13]. Similar to the procedures of the 1-Stroke testing, data collection occurred in the rowing team’s training facility. The athletes were split into two groups, and within each group, the athletes performed the testing procedures simultaneously. The participants completed a warm-up at a resistance setting of 1 on the control dial for 5 min. After a short rest of 180 s, the participants proceeded with the 3-min all-out row. During the 3-min rowing test, the ergometer was programmed at its maximum damper setting (ten on the resistance control dial). To prevent pacing during the test, participants were blinded to the rowing ergometer’s monitor information and the elapsed time. To ensure an all-out effort, participants were instructed to maintain their stroke rates as high as possible for the duration of the test. Thirty-second average power outputs (watts) were recorded in the Concept II memory card and transferred to a laboratory computer. The CP_3-min_ value was calculated as the average power output for the final 30 s of the test.

#### 2.2.6. Rowing Ergometer Performance

The 6K and 2K simulated rows were done on separate days with at least 72 h between tests. In addition to the 2K, the 6K was part of the team’s normal performance testing to establish the athletes’ aerobic fitness levels during the preseason. Performance tests were done on Concept II rowing ergometers (“time-trial” mode). Testing took place during the team’s morning training session between 6:00 and 9:00 AM. Data collection for 6K and 2K took place inside the rowing team’s training facility, in an area with multiple rowing ergometers that allowed for large scale testing. Prior to the start of the time-trials, the athletes warmed up for 30 min as a team. Upon completion of the 30 min warm up, athletes were given approximately five minutes to get water and set up the ergometers. The ergometer was programmed at a damper setting of “3–4” on the control dial. The athletes were instructed to “simulate an on-water race”, starting with an initial spurt followed by a constant pace at their preferred stroke rate. The dependent variables of interest to reflect time-trial performance were total completion time (6K_total_ and 2K_total_) and speed quantified through average 500 m split time (6K_split_ and 2K_split_), as split time has been shown to be a valuable tool in gauging performance outcomes and tracking improvement throughout training [26].

### 2.3. Statistical Analysis

A Shapiro–Wilk test was used to determine if experimental variables were normally distributed. Pearson product-moment correlation coefficients (*r*) were calculated to assess the association between experimental and performance variables. Correlation values between 0 and 0.30 were considered small, 0.31 to 0.49 was moderate, 0.50 to 0.69 was large, 0.70 to 0.89 was very large, and 0.90 to 1.00 was near-perfect [27]. The correlation coefficients between $\dot{V}O$_2peak_ and rowing performances were compared with those between CP_3-min_ and the rowing performances, after the Fisher r-to-z transformation was applied to each correlation coefficient. Similarly, the z transformed correlation coefficients between peak power from WAnT and the rowing performances were compared with those between 1-Stroke and the rowing performances [28,29]. Cohen’s *q* was used to determine the effect size (ES) of the magnitude of difference between strength of correlations; classifications for interpretation were as follows: <0.1 was no effect, 0.1–0.3 was a small effect, 0.3–0.5 was an intermediate effect, and >0.5 was large effect [30]. Stepwise linear regressions were analyzed to determine the prediction power of the field- and laboratory-based tests and relative contribution of each towards 6K and 2K rowing ergometer performance. Unless otherwise stated, all data were presented as mean ± standard deviation (*M* ± *SD*) and statistical significance was accepted at *p* < 0.05.

## 3. Results

**Figure 1:**
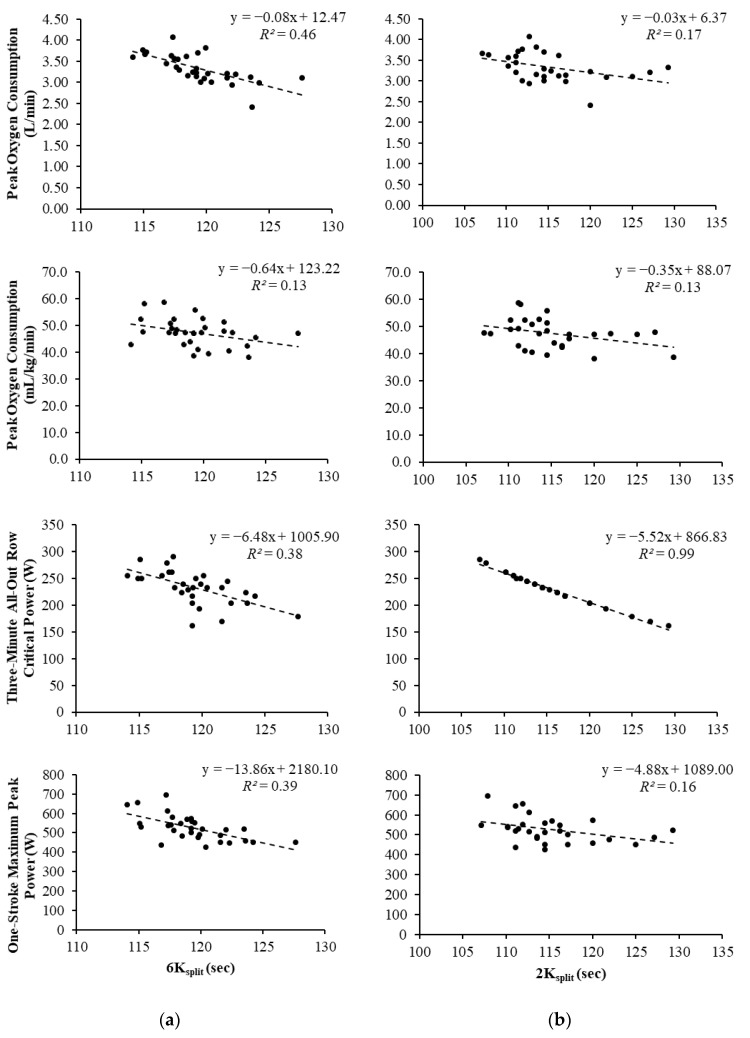
Scatterplots representing the relationship between 6K/2K split times and fitness tests. (**a**) 6K_split_ = 500 m split time for 6000 m row; (**b**) 2K_split_ = 500 m split time for 2000 m row.

**Figure 2:**
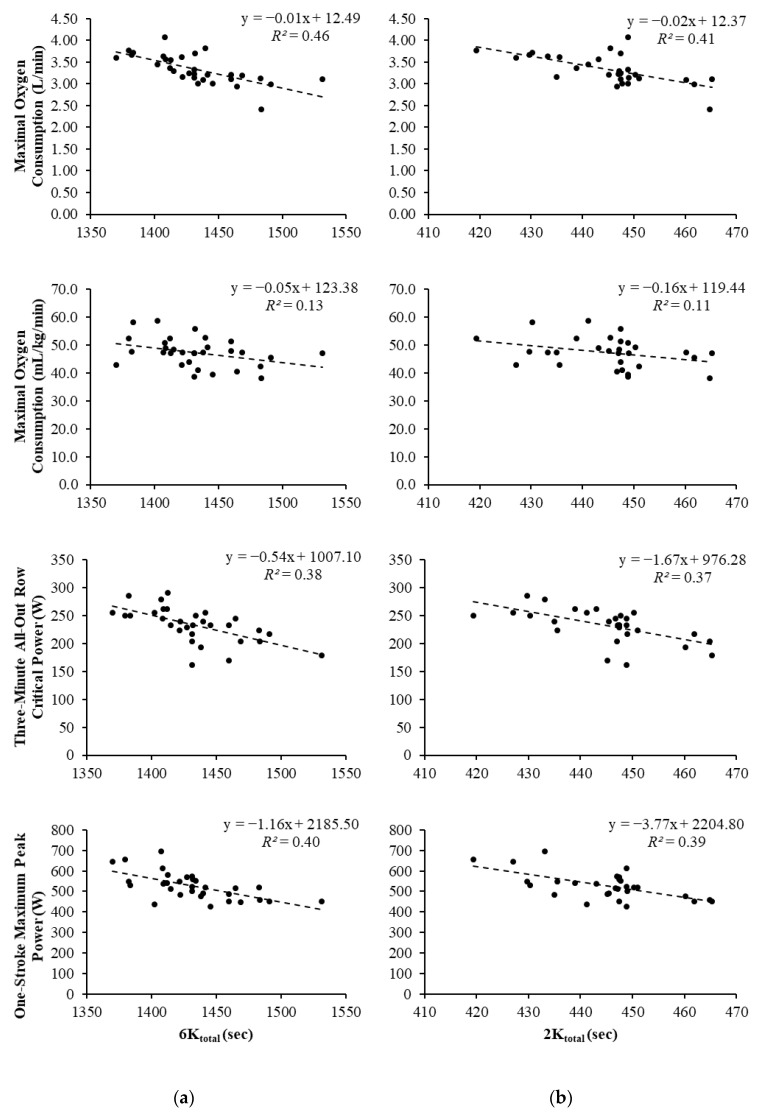
Scatterplots representing the relationship between 6K/2K completion times and fitness tests. (**a**) 6K_total_ = 6000 m row completion time; (**b**) 2K_total_ = 2000 m row completion time.

A total of 31 athletes participated in this study; however, two were unable to complete the 2K due to injury and illness. All bivariate correlation coefficients between rowing performance and fitness testing variables are displayed in Table 1 and Table 2. Scatterplots depicting relationships between fitness assessments and rowing ergometer performance are displayed in Figure 1 and Figure 2.

### 3.1. 6000 m and 2000 m Completion Times

Absolute $\dot{V}O$_2peak_ values displayed significant, large correlations with 6K_total_ and 2K_total_. The association between relative $\dot{V}O$_2peak_ and 6K_total_ was significant and considered moderate, while the correlation with 2K_total_ was non-significant and moderate. Significant, large correlations were observed between CP_3-min_ and 6K_total_ and 2K_total_. In relation to 6K_total_ rowing performance, the Fisher r-to-z transformation found no significant difference between relative $\dot{V}O$_2peak_ and CP_3-min_ correlation values (*z* = 0.40, *p* = 0.69) and a “small” ES (*q* = 0.11). For 2K_total_, no significant difference was observed (*z* = 0.19, *p* = 0.849) with “no effect” (*q* = 0.05). Significant, large associations were observed between 1-Stroke and 6K_total_ and 2K_total_, while WAnT demonstrated small, non-significant correlations with 6K_total_ and 2K_total_.

Relative $\dot{V}O$_2peak_ was not utilized in the regression analysis due to the stronger correlations being displayed by absolute $\dot{V}O$_2peak_. In both the 6K_total_ and 2K_total_ regressions, WAnT and 1-Stroke were excluded from the models, with absolute $\dot{V}O$_2peak_ and CP_3-min_ being retained. For the 6K_total_ regression, when absolute $\dot{V}O$_2peak_ was used as the sole predictor, it accounted for ~46% of the variance in completion time that could be predicted (*R* = 0.68, *R*^2^ = 0.46, *p* < 0.001). The addition of CP_3-min_ for the second model accounted for ~58% of the variance (*R* = 0.76, *R*^2^ = 0.58), causing a significant change in prediction power (*p* = 0.008). The squared semipartial correlation (*sr*^2^) that estimated how much variance in 6K_total_ was uniquely predictable from absolute $\dot{V}O$_2peak_ was *sr*^2^ = 0.201, meaning ~20% of the variance in 6K_total_ performance was uniquely predictable from $\dot{V}O$_2peak_ when CP_3-min_ was controlled. The *sr*^2^ for CP_3-min_ (controlling for $\dot{V}O$_2peak_) was 0.121. Thus, CP_3-min_ uniquely predicted ~12% of the variance in 6Ktotal when $\dot{V}O$_2peak_ was controlled. The predictive equation was as follows: 6Ktotal = 1714.69 + (−53.49 × $\dot{V}O$_2peak_) + (−0.45 × CP_3-min_).

For the 2K_total_ regression, when absolute $\dot{V}O$_2peak_ was used as the sole predictor, it accounted for about 41% of the variance in completion time could be predicted (*R* = 0.64, *R*^2^ = 0.41, *p* < 0.001). The addition of CP_3-min_ for the second model accounted for 54% of the variance (*R* = 0.74, *R*^2^ = 0.54), causing a significant change in prediction power (*p* = 0.011). The *sr*^2^ for $\dot{V}O$_2peak_ was 0.17, predicting ~17% of the variance in 2K_total_. The *sr*^2^ for CP_3-min_ was 0.116, predicting ~12% of the variance. The predictive equation was as follows: 2K_total_ = 527.45 + (−14.57 × $\dot{V}O$_2peak_) + (−0.15 × CP_3-min_).

### 3.2. 6000 m and 2000 m Split Times

Absolute $\dot{V}O$_2peak_ values displayed a significant large correlation with 6K_split_ and a moderate correlation with 2K_split_. The association between relative $\dot{V}O$_2peak_ and 6K_split_ was significant and considered moderate, while the correlation with 2K_total_ was non-significant and moderate. CP_3-min_ values displayed a significant, large correlation with 6K_split_ and a near-perfect association with 2K_split_. In relation to 6K_plit_ rowing performance, the Fisher r-to-z transformation found no significant difference between relative $\dot{V}O$_2peak_ and CP_3-min_ correlation values (*z* = 0.40, *p* = 0.689) and a “small” ES (*q* = 0.107). For 2K_split_, a significant difference was observed (*z* = −8.53, *p* < 0.001) with a “large” ES (*q* = 2.367). Moreover, 1-Stroke power output values demonstrated a significant, large relationship with 6K_split_ and a significant, moderate correlation with 2K_split_, while both WAnT demonstrated small, non-significant correlations with 6K_split_ and 2K_split_.

For the 6K_split_ regression, when absolute $\dot{V}O$_2peak_ was used as the sole predictor, it accounted for about 46% of the variance in completion time that could be predicted (*R* = 0.679, *R*^2^ = 0.461, *p* < 0.001). The addition of CP_3-min_ for the second model accounted for 58% of the variance (*R* = 0.762, *R*^2^ = 0.581), causing a significant change in prediction power (*p* = 0.008). The *sr*^2^ for $\dot{V}O$_2peak_ was 0.202, predicting about 20% of the variance in 6K_split_. The *sr*^2^ for CP_3-min_ was 0.120, predicting about 12% of the variance. The predictive equation was as follows: 6K_split_ = 142.892 + (−4.475 × $\dot{V}O$_2peak_) + (−0.037 × CP_3-min_). For the 2K_split_ regression, absolute $\dot{V}O$_2peak_, WAnT, and 1-Stroke variables were excluded in the analysis. As the sole predictor, CP_3-min_ accounted for about 99% of the variance in split time that could be predicted (*R* = 0.993, *R*^2^ = 0.987, *p* < 0.001). The predictive equation was as follows: 2K_split_ = 156.597 + (−0.179 × CP_3-min_).

## 4. Discussion

The purpose of this study was to examine the association of CP_3-min_ and peak power from 1-Stroke with traditional laboratory-based fitness assessments and with 6K and 2K rowing ergometer performance. Results of the study showed that $\dot{V}O$_2peak_, CP_3-min,_ and 1-Stroke demonstrate strong relationships with rowing ergometer performance values, and that absolute $\dot{V}O$_2peak_ and CP_3-min_ produce significant prediction models for of both total completion and split times. Additionally, no significant difference was observed between the correlations of CP_3-min_ and absolute $\dot{V}O$_2peak_ for 6K_total_, 2K_total,_ and 6K_split_ rowing times; however, a significantly stronger relationship was observed between CP_3-min_ and 2K_split_. Finally, WAnT peak power outputs were not significantly associated with rowing ergometer performance.

### 4.1. Rationale for Methodology

Certain testing procedures were chosen at the request of the team’s coaching staff (e.g., treadmill GXT and cycle WAnT). Previous research investigating the relationship of $\dot{V}O$_2peak_ from a GXT and power output from the WAnT with rowing performance have done so using rowing ergometer-based testing procedures [20,31], in order to better simulate on-water racing scenarios. The rationale for the utilization of the current study’s varying modality approach was to evaluate fitness parameters independent of technical rowing skill and expertise; however, because the tests of the current study differed in primary muscle groups utilized and the capabilities of those muscles, comparison between methods is somewhat difficult. Excluding the 1-Stroke test, the testing procedures used were novel to all participating athletes, which in theory allowed for a more equivalent comparison of fitness assessment scores from first-year athletes to the more experienced seniors. Though some methodological limitations exist, there is still knowledge to be gained from the results for practical application in amongst collegiate teams and athletes.

### 4.2. Aerobic Fitness Assessments

It is well-established that success in endurance-based sports (e.g., rowing) is strongly associated with high $\dot{V}O$_2max_, largely due to its setting of the upper limit for steady-state oxygen consumption [2,3,4]. In the present study, $\dot{V}O$_2peak_ was acquired instead of a true max, which requires a plateau of oxygen uptake during the initial test followed by a verification test after 20-min of rest [32]. Time constraints and limited access to the current study’s athletes made true $\dot{V}O$_2max_ testing impossible. However, $\dot{V}O$_2peak_ displayed large correlations with rowing ergometer performance, and it accounted for significant portions of the variance seen in rowing performance measures. Although the efficacy of traditional laboratory-based aerobic power testing is clear, the impracticality of proper assessment, highlighted by the limitations of the current study’s procedures, further illustrates the need for field-based tests.

Theoretically, this same upper limit for steady-state oxygen consumption found with GXT testing can also be derived through CP assessment [8,33]. Previous research has observed associations between critical power values derived from cycle ergometer 3-min all-out testing and $\dot{V}O$_2peak_ [34], which falls in line with the results of the current study. The findings of the present study build on this foundational research by demonstrating moderate correlations between $\dot{V}O$_2peak_ from a treadmill GXT and CP_3-min_ (*r* = 0.45). Additionally, to the best of our knowledge, this was the first study to demonstrate large correlations between CP_3-min_ and rowing performance. Based on these results, practitioners may be able to use CP_3-min_ as a field-based measure of aerobic fitness and a predictor of rowing ergometer performance. One shortcoming of the current study was that oxygen consumption was not measured during the CP_3-min_; this additional physiological data may further explain the significant relationship found between CP_3-min_ and $\dot{V}O$_2peak_. Additionally, although the results of the current study hold value, controversy behind the validity of the 3-min all-out test still remains [35]. Future studies should look to incorporate $\dot{V}O$_2_ testing during both the 3-min all-out and traditional multi-bout CP testing, as well as rowing ergometer time-trials, to gather additional information on oxygen consumption patterns and further validate the use of CP_3-min_ to gauge aerobic capacity.

### 4.3. Anaerobic Fitness Assessments

In the present study, no significant correlations were found between WAnT metrics and 6K or 2K simulated race times, which directly contradicts previous literature. Koutedakis and Sharp [36] used a modified Wingate protocol to assess upper body anaerobic power and demonstrated strong correlations with strength and prediction of performance [36]. Riechmann et al. [37] found that 75.7% of the variation in 2K indoor rowing performance time was predicted by peak power in a rowing WAnT, while $\dot{V}O$_2max_ and fatigue assessed during the WAnT explained an additional 12.1% and 8.2% of the variance, respectively [37]. The weak relationship observed in the current study may be attributed to the nature of the test and modality utilized. A cycle-based WAnT only assesses lower-body anaerobic power, while rowing involves full-body mechanics. Previous studies have shown that the modified WAnT on a Concept II rowing displayed strong correlations with rowing performance [5,6,37] and produced significantly higher peak power outputs than cycle testing [31]. Conversely, Cerasola et al. [38] found a near-perfect association between 2K performance and cycle WAnT peak power (*r* = 0.90) [38]. Future research should look to further compare the relationship between cycle- and rowing ergometer-based WAnT with rowing performance.

Peak power from the 1-Stroke displayed significant associations with both 6K and 2K. Additionally, 1-Stroke maximum power outputs were found to be significantly related to both absolute $\dot{V}O$_2peak_ and CP_3-min_ but not significantly associated with WAnT. It was our assumption that 1-Stroke is a metabolically anaerobic test due to the short duration of the activity (≤1 s); however, larger relations were found with aerobic assessments. Due to the conflicting nature of this novel test, practitioners should be cautious when implementing or interpreting results. Future research should examine 1-Stroke’s association with validated measures of muscular strength and power in addition to both the WAnT and modified WAnT.

## 5. Conclusions

Laboratory-based tests are not easily accomplished, especially for teams who do not have readily available access to an exercise physiology laboratory with the proper equipment. The CP_3-min_ and 1-Stroke tests may provide further insight into gauging aerobic and anaerobic power and predicting 6K and 2K performance. Practitioners with the appropriate tools available to them should look to incorporate these field tests to gather a better understanding of athlete fitness capabilities.

## Figures and Tables

**Table 1:** Correlations with 6K Rowing Performance.

	*n*	*M* ± *SD*	6K_total_		6K_split_	
			*r*	*p*	*r*	*p*
6K_total_ (s)	31	1432.73 ± 35.71	-	-	-	-
6K_split_ (s)	31	119.35 ± 2.98	-	-	-	-
$\dot{V}O$_2peak_ (L/min)	31	3.33 ± 0.34	−0.36	0.044	−0.37	0.044
$\dot{V}O$_2peak_ (mL/kg/min)	31	47.26 ± 5.21	−0.68	<0.001	−0.68	<0.001
WAnT (watts)	31	753.74 ± 137.34	−0.11	0.546	−0.11	0.555
CP_3-min_ (watts)	31	2 32.61 ± 31.27	−0.62	<0.001	−0.62	<0.001
1-Stroke (watts)	31	526.06 ± 65.77	−0.63	<0.001	−0.63	<0.001

Notes: 6K = 6000 m ergometer row; *n* = number of participants; *M* ± *SD* = mean ± standard deviation; $\dot{V}O$_2peak_ = peak oxygen uptake; CP_3-min_ = critical power from 3-min all-out test; 1-Stroke = one-stroke maximum test; WAnT = Wingate anaerobic test.

**Table 2:** Correlations with 2K Rowing Performance.

	*n*	*M* ± *SD*	2K_total_		2K_split_	
			*r*	*p*	*r*	*p*
2K_total_ (s)	29	444.96 ± 10.83	-	-	-	-
2K_split_ (s)	29	115.16 ± 5.38	-	-	-	-
$\dot{V}O$_2peak_ (L/min)	29	3.33 ± 0.34	−0.33	0.084	−0.33	0.069
$\dot{V}O$_2peak_ (mL/kg/min)	29	47.27 ± 5.39	−0.64	<0.001	−0.43	0.016
WAnT (watts)	29	760.35 ± 131.09	−0.06	0.753	−0.05	0.782
CP_3-min_ (watts)	29	231.62 ± 29.90	−0.61	<0.001	−0.99	<0.001
1-Stroke (watts)	29	526.83 ± 65.68	−0.62	<0.001	−0.44	0.013

Notes: 2K = 2000 m ergometer row; *n* = number of participants; *M* ± *SD* = mean ± standard deviation; $\dot{V}O$_2peak_ = peak oxygen uptake; CP_3-min_ = critical power from 3-min all-out test; 1-Stroke = one-stroke maximum test; WAnT = Wingate anaerobic test.
